# Extreme ecchymoses in a migraine patient using concomitant treatment with calcitonin gene-related peptide receptor antibodies and fish oil supplements: a case report

**DOI:** 10.1186/s12883-021-02294-6

**Published:** 2021-07-02

**Authors:** C. K. Cullum, M. K. Olsen, H. B. Kocadag, M. Ashina, F. M. Amin

**Affiliations:** Danish Headache Center, Department of Neurology, Rigshospitalet Glostrup, University of Copenhagen, Valdemar Hansens Vej 5, 2600 Glostrup, Denmark

**Keywords:** Migraine, Erenumab, CGRP monoclonal antibody, Fish oil, Ecchymosis, Bleeding, Hemostasis

## Abstract

**Background:**

Erenumab, a monoclonal antibody against the calcitonin gene-related peptide (CGRP) receptor, is registered for migraine prevention. Compared to other conventional migraine prevention medicines (i.e. topiramate, betablockers and amitriptyline) erenumab has better tolerability. Impaired hemostasis has not been reported previously. Here, we report the first case of an increased tendency to bruise in a migraine patient treated with erenumab.

**Case presentation:**

A 41-year old female migraine patient was treated with erenumab for 12 months, which led to a significant reduction of headache and migraine days. Three months after treatment start, she experienced increased tendency to bruise leading to extreme ecchymosis after 4 months treatment. Platelet counts and aggregation, thromboelastography, activated partial thromboplastin time (APTT) and international normalized ratio (INR) were all normal. Thorough interview revealed intake of fish oil supplements for many years prior to treatment. The increased tendency to bruise subsided after discontinuation of fish oil supplements.

**Conclusion:**

The combination of fish oil supplements and erenumab may cause increased tendency to bruise. Erenumab has no effect on the platelets per se but may cause impaired wound healing by suppression of CGRP. Thus, small and unnoticeable bruises may be aggravated instead in patients with tendency to bruise caused by for instance fish oil supplements.

## Background

Treatment with monoclonal antibodies against calcitonin gene-related peptide (CGRP) is a new generation of mechanism-based preventive antimigraine therapy. CGRP is a vasoactive peptide found naturally in the organism, including the trigeminovascular system. Intravenous infusion of CGRP causes migraine attacks [1]. Erenumab, a CGRP receptor antibody, is effective against chronic migraine and has relatively few side effects [2] compared to the conventional antimigraine drugs. The most common adverse events include constipation, muscle spasms itching, injection site pain, nasopharyngitis, and upper respiratory tract infections [3]. Here, we present the first case of increased tendency of ecchymosis in a chronic migraine patient treated with a monoclonal CGRP receptor antibody, erenumab.

## Case presentation

In November 2019, a 41-year old Caucasian female with chronic migraine, was started on preventive antimigraine treatment with 140 mg subcutaneous erenumab every 4 weeks. At the time of treatment start the patient had 16 headache days a month of which 12 was migraine days. The patient used either frovatriptan, sumatriptan injections or eletriptan with successful pain mitigation between 3–8 days a month. She suffered from migraine without aura since childhood but was otherwise healthy, except rare occasional small unnoticeable bruises during the past 4–5 years without relationship to traumas. She had no family history of hemostasis disorders. She had previously been treated with various antimigraine prophylactics, including antihypertensive drugs (candesartan, lisinopril, metoprolol), anticonvulsants (topiramate, lamotrigine) as well as botulinum toxin type A, flunarizine, riboflavin, and magnesium without effective reduction in migraine days.

At the 3-month follow-up there was a significant reduction in headache (50%) and migraine (50%) days at month 3. The patient reported mild constipation as well as an increased tendency to bruise without the patient having any recollection of any traumas. The spontaneous bruising occurred primarily on her lower legs and thighs, but also on her upper arms and had no spatial relation to the injections site of the erenumab as the patient reported she always injected in her stomach. The patient reported no bruising in the injection site. Moreover, the patient had no fever or general aches. Blood samples showed normal platelet count (198 × 10^9^/L) and aggregation, thromboelastography, APTT (29 secs) and INR (1.0). The patient denied any trauma, tendency to bleed from mucous membranes, affected menstrual cycle, or use of aspirin, NSAIDs or other blood thinners. We advised the patient to pause erenumab treatment, but she wanted to continue because of the excellent effect on her headache and migraine. Thus, she continued with erenumab treatment, but was lowered to a dose of 70 mg as part of standard treatment procedure at the Danish Headache Center.

**Fig. 1:**
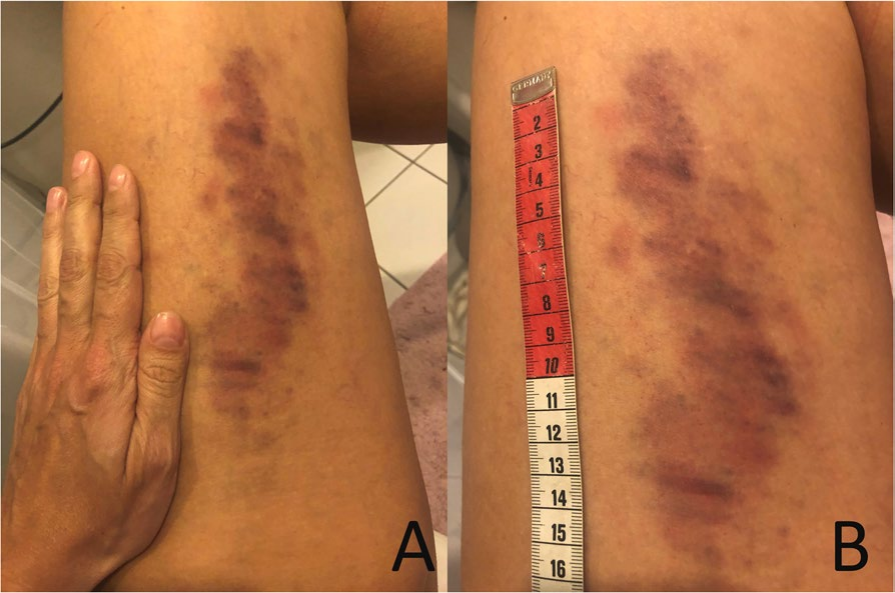
Ecchymoses on the patient’s upper left thigh with **A** patient’s hand for scale, and **B** with a ruler

After another 5 weeks, the patient contacted the headache center because of extreme aggravation of the bruising tendency as she woke up with a 16 cm × 8 cm ecchymosis on each thigh (Fig. 1).

Again, she was thoroughly interviewed and denied trauma, local pain, itching etc. Review of her use of medication, revealed a daily use of fish oil supplements for many years which the patient did not regard as medication. She reassured, that she had never experienced similar tendency to bruise. As the fish oil was suspected of having an interaction with erenumab causing hemostatic disturbances, the patient was advised to immediately stop the use of fish oil supplement, as she was not willing to discontinue erenumab. The patient was discussed with a hematologist who advised a referral to the dermatologists for skin biopsy if no improvement was seen after cessation of the fish oil. However, the patient had no other clinical symptoms of a potential autoimmune vasculitis.

**Fig. 2:**
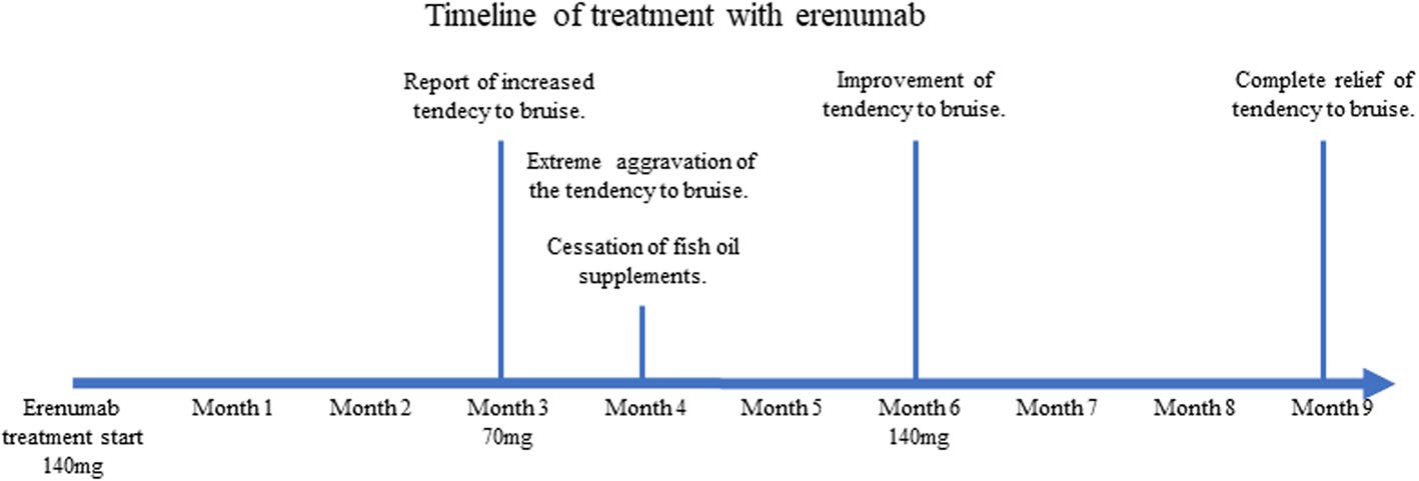
Timeline of treatment period, showing dosage of erenumab treatment and clinical events

Improvement of the tendency to bruise was reported after 6-months, where the erenumab dose was increased from 70 to 140 mg again because of better efficacy of 140 mg in the first 3 months. There was no increased tendency to bruise after 9 and 12 months and the patient continued to have satisfactory effect of 140 mg erenumab every 4 weeks (For timeline overview see Fig. 2).

## Discussion and conclusion

Ecchymosis or hemostatic disturbances associated with CGRP receptor antibody treatment have previously not been reported in pre-marketing or the subsequent post marketing studies [3]. By contrast, the omega-3 polysaturated fatty acids from fish oil are associated with slightly increased risk of bleeding events [4]. In otherwise healthy subjects, fish oil supplements may cause reduced platelet aggregation [5] and adhesion [6]. The present patient had used fish oil supplements for at least 4 years without experiencing any noticeable bruises, before start of erenumab treatment. Because of normal hemostatic factors in blood and cessation of the increased ecchymosis tendency after discontinuation of fish oil supplements, an interaction between erenumab and fish oil supplements may be suspected in our patient. The exact role of erenumab in hemostasis is unknown. A toxicology study reports no effect of erenumab per se on the platelets [7]. Preclinical research in animal and human tissue in vitro have shown that CGRP plays a favorable role in wound healing [8]. A recent case story reported a female migraine patient with impaired wound healing after mild traumas during treatment with erenumab [9]. Bruises are flat patches on the skin caused by blood extravasation to the tissue from ruptured capillaries either caused by traumas or because of underlying coagulopathy.

Usually, the underlying damage behind bruises heal rapidly, but because of the possible suppression of CGRP function, the healing may be delayed, which cause large extravasation of blood. This was most likely the reason for the large ecchymosis in our patient, rather than a direct interaction between erenumab and the fish oil supplements. Likewise, it is unlikely that erenumab per se causes increased risk of bleeding. Possible explanation for not reporting bruises as side effects or complication may be that the tendency to bruise is quite common, they cause no pain and disappear after some days. Moreover, the phase 2 and 3 clinical randomized trials usually exclude patients with the need of concomitant anticoagulant and antiplatelet drugs for prevention of cardio- or cerebrovascular disease.

Based on this case report, we suggest extra caution in patients with tendency to bruise or known coagulopathy when therapy with CGRP receptor antibody is initiated. The risk of large bruises may be increased in patients who use concomitant fish oil supplements or other blood thinners, including prophylactic anticoagulants or platelet inhibitors in vascular stigmatized patients. In addition, extra caution should be practiced in relation to surgical procedures in patients receiving CGRP receptor antibodies. Future studies may clarify if all types of CGRP suppressing drugs increase the risk of frequent and larger bruises.

## Clinical implications

1. We present a migraine patient treated with erenumab while taking fish oil supplements who developed increasing aggravation of bruising tendency over several months of treatment, despite reduction of erenumab dosage. Cessation of fish oil supplements improved bruising tendency despite reverting to full dosage erenumab.
2. This is the first reported case of concomitant erenumab and fish oil causing severe spontaneous bruising in a patient with no known coagulopathy.
3. Caution is advised when prescribing erenumab to patients with a tendency to bruise easily, who have pre-existing coagulopathies or are receiving blood thinners such as fish oil, platelet inhibitors or anticoagulants due to a possible increased risk of severe bruising.

## Data Availability

Data sharing is not applicable to this article as no datasets were generated or analysed during the current study.

## Abbreviations

CGRP: Calcitonin gene-related peptide
APTT: Activated partial thromboplastin time
INR: International normalized ratio
